# A qualitative exploration of the provision and prioritisation of smoking cessation support to patient carers in a paediatric ward in Australia

**DOI:** 10.1186/s12912-022-01010-0

**Published:** 2022-08-16

**Authors:** Sukoluhle Moyo, Marita Hefler, Kristin V. Carson-Chahhoud, David P Thomas

**Affiliations:** 1 Menzies School of Health Research, Charles Darwin University, Alice Springs, NT Australia; 2 Menzies School of Health Research, Charles Darwin University, PO Box 41096, Casuarina, Darwin, NT 0811 Australia; 3 University of South Australia, GPO Box 2471, Adelaide, South Australia 5001 Australia

**Keywords:** Paediatric, Aboriginal, Smoking, Second hand-smoke, Hospital

## Abstract

**Background:**

Hospitalisation of a child is a unique opportunity for health staff to offer smoking cessation support; that is screening for carer smoking status, discussing cessation and providing interventions to carers who smoke. This has the potential to reduce the child’s exposure to second-hand smoke, and in turn tobacco related illnesses in children. However, these interventions are not always offered in paediatric wards. The aim of this study was to explore the provision and prioritisation of smoking cessation support to patient carers in a paediatric ward with a high proportion of Aboriginal patients and carers in a regional area of Australia’s Northern Territory.

**Methods:**

This is a qualitative descriptive study of data collected through semi-structured interviews with 19 health staff. The interviews were audio recorded and transcribed verbatim. Thematic analysis was performed on the transcripts.

**Results:**

We found low prioritisation of addressing carer smoking due to, a lack of systems and procedures to screen for smoking and provide quitting advice and unclear systems for providing more detailed cessation support to carers. Staff were demotivated by the lack of clear referral pathways. There were gaps in skills and knowledge, and health staff expressed a need for training opportunities in smoking cessation.

**Conclusion:**

Health staff perceived they would provide more cessation support if there was a systematic approach with evidence-based resources for smoking cessation. These resources would include guidelines and clinical record systems with screening tools, clear action plans and referral pathways to guide clinical practice. Health staff requested support to identify existing training opportunities on smoking cessation.

## Background

Daily smoking prevalence among Aboriginal and Torres Strait Islander people aged 15 years and over has fallen from 41% in 2012–2013, to 37% in 2018–19 [1]. Smoking prevalence among Aboriginal and Torres Strait Islander people is higher than in the overall Australian population and is closely linked to the historical and ongoing impacts of colonisation including trauma and racism [2].

Smoke-free public spaces legislation has been widely implemented in Australia to prevent exposure to second-hand smoke (SHS) [3]. Smoke-free public spaces legislation is associated with an increase in smoke-free homes [4, 5], which in turn contributes to declines in exposure of children to SHS and childhood hospital admissions [5]. Despite these interventions, the proportion of Aboriginal and Torres Strait Islander children aged 0–14 years of age exposed to SHS in their home continues to be significantly higher than non-Indigenous children [6]. The proportion of Aboriginal and Torres Strait Islander children aged 0–14 years who lived in a household where smoking occurred inside the house declined from 28% in 2004 to 21% in 2008, compared to a decline from 9 to 7% for non-Indigenous children in the same period [6].

SHS increases the risk of child mortality, and also causes and exacerbates a range of paediatric illnesses such as asthma, respiratory infections and otitis media [7–10]. From 2013 to 2015 the annual rate of hospitalisation for respiratory illnesses in Aboriginal and Torres Strait Islander children aged 0–4 years was 78 per 1,000 population compared with 48 per 1,000 population for non-Indigenous children [9]. The most effective way to protect children from SHS exposure in the home is for carers to quit smoking [11].

Hospitalisation of children in paediatric wards presents an opportunity for health staff to screen for smoking status, advise and support carers who smoke to quit in order to protect children from SHS, and in turn contribute to reducing SHS-related illnesses. Unlike the large body of evidence around inpatient smoking cessation programs for adults [12], there is little knowledge about experiences of health workers providing smoking cessation programs targeting carers of children within paediatric inpatient settings [10, 13, 14].

This paper aimed to explore the provision of smoking cessation support to patient carers in a paediatric ward of a regional hospital in the Northern Territory of Australia with a large proportion of Aboriginal patients and carers.

## Methods

This was a descriptive qualitative study using data collected as part of a larger PhD project undertaken by the first author that examined the role of health staff in delivering targeted smoking cessation programs in paediatric health care settings to reduce SHS exposure. One component of this project has already been published, examining miscommunication and misperceptions between health staff and Indigenous carers about raising the topic of smoking cessation in the paediatric ward [15]. A descriptive thematic analysis using an inductive approach, as described by Braun and Clarke [16], was used to explore barriers and enablers impacting health staff provision of smoking cessation support to patient carers in the paediatric ward.

The initial thematic analysis suggested that the data be described in two separate papers. The first paper was analysing data related to communication and has been published [15]. This paper analyses remaining data, not previously examined in the original publication. A third paper will describe data collected during initial stages of translating the evidence we produced from our first two papers, into the clinical setting to improve policies and practices at the participating hospital. Research methodology and associated tools were designed to deliver three original research outputs, including questions build into the moderator guide specific to each paper produced.

Thematic analysis has the advantage that it can map out the range and strengths of views across a selection of participants [17]. We have used an inductive and data driven approach which helped to minimise the bias that could be caused by the researchers’ analytic preconceptions [16]. There was aspects of an inductive approach as well as the first researcher came fully emersed in the knowledge about existing issues related to carer smoking support, and used a theory-based approach to pre-define the context and issues to be addressed by this study [16, 18].

The first researcher was a paediatric clinical nurse educator in the hospital, which involved working directly with patients and health staff at the time while conducting the research.

The first researcher is a woman and originally from Zimbabwean background. She has lived in Australia since 2012 and worked at the study hospital since then. The other authors are all Australian-born of Anglo-Celtic and/or European family background, with English as a first language, and work in Aboriginal and Torres Strait Islander health research.

### Study participant selection and recruitment

Medical staff (consultants, registrars, resident medical officer and medical students) and nurses (senior nurses and student nurses) were directly approached to participate in the study. Purposive sampling was used to ensure maximum variation of staff roles and backgrounds [19, 20], including health staff with a managerial role (HSMR) who were involved in formulating policies and strategic decision making, and staff who have direct contact with carers. Some HSMR also had a clinical role. The selection of study participants targeted health staff with a medical or nursing background with or without a managerial role, who routinely provided care to children admitted in the Paediatric Ward [20]). We excluded nursing and medical staff in the Emergency Department and other health staff who worked in the paediatric ward such as the allied health team.

### Data collection

A discussion guide was used to direct in-depth semi-structured interviews. The guide included broad thematic questions and prompts about health staff prioritisation of smoking cessation support for carers, knowledge of health consequences of SHS, and barriers and enablers for providing carer smoking cessation support in the paediatric ward. The interview questions were open-ended and allowed the researcher to derive themes, both deductively guided by existing literature, and inductively from the participants’ lived experiences of providing care to children and their carers. The first researcher also used observation and taking field notes in her day-to-day practice as other sources of information to triangulate data collected during interviews and assist with analysis.

The first researcher conducted all interviews in English in a private room of the paediatric ward between October 2018 to November 2019, with only the participant present in the room. The interviews were audio-recorded and transcribed verbatim. Names, precise job titles and other personal details were removed to preserve anonymity. All initial codes were generated from the first 13 interviews.

### Data analysis

The first and second researchers familiarised themselves with the data from reading the verbatim transcripts and memos, and generated initial codes that based on key issues and patterns of behaviour among participants [16]. The codes were then organised into overarching and broad themes. An iterative process was used to review and refine the broad themes into dense themes. All the researchers contributed to formulating the final themes which then culminated in the production of a scholarly report of the analysis.

### Ethical considerations

Approval was granted by the NT Department of Health & Menzies School of Human Research Ethics Committee, Central Australian Human Research Ethics Committee, Charles Darwin University Human Research Ethics Committee and the participating hospital.

There was potential for staff to feel compelled to participate, particularly as the first researcher’s position was a more senior role than some of the participants. This potential risk was mitigated by providing a detailed participant information sheet explaining the goals of the research and that participation was voluntary and giving assurance that their responses would be treated with confidentiality and would not affect work relationships. Participants all gave signed informed consent.

## Results

The researcher directly approached 26 ward health staff. Seven declined to participate, and the researcher did not seek reasons for non-participation. Of the 19 health workers who participated in this study, 14 were nurses and five were doctors (Table 1). Two of the nurses and four of the doctors had both managerial and clinical roles. Quotes are presented here with identifiers for participants’ backgrounds as either health staff (HS) or HSMR. Interviews lasted from 25 to 80 min.

**Table 1:** Characteristics of Study Participants

		Non-managerial Health staff		Managerial health staff
	Total	Temporary	Permanent	
Nurse	14	3	11	3
Doctor	5	1	4	4

Smoking cessation was a low priority among paediatric health staff in our study, who felt poorly equipped to address carers’ smoking unless it was directly related to the presenting health issue of the child. A systematic approach, with guidelines and clinical record systems, could offer opportunities to better support health staff to improve clinical practice.

### Low prioritisation of carer smoking

Although health staff acknowledged that carer cessation support [screening for carer smoking status, discussing cessation and providing interventions], needed to be prioritised, it was a relatively low priority compared to many health and social issues facing the child, carer and broader family. External factors that were higher priorities for health staff in the paediatric ward included mental health, housing, child safety and domestic violence.

*There are just so many things to explore with families. So, if you’re exploring mental health, domestic violence, appropriate housing, child safety with families, there are lots to explore. And I suppose the reason smoking gets pushed down there is that it’s a risk factor, but compared to some of the risk factors around, it’s…how would you say it? It’s not as often, it’s an acute factor, but it’s not as acute a threat ethically, I suppose. All the others [issues] seem to jump up and wave red flags in front of us where maybe smoking does not. HSMR 2*

*I do think it is important that we do address these issues and make it a priority, but sometimes it’s not the most important thing at the time. HS 1*

Presenting illnesses were considered to be a more urgent medical issue than the latent effects of SHS exposure that did not necessarily pose an immediate threat to the life of the child.

*The medical team might be focused entirely on the acute issue, especially if the child is unwell. HSMR 2*

The exception to the low priority given to offering cessation support was carers whose children presented to hospital with respiratory illnesses and other conditions that may have had a direct link to smoking. The usual clinical practice was that staff said they did not routinely screen for carers’ tobacco use when a child’s diagnosis did not have a clear link to smoking. Health staff felt that there was a need for them to see a connection between the child’s presenting health problem and tobacco use to prioritise raising this topic. If there was no clear link, then they did not think it was appropriate to raise smoking. Low prioritisation in talking to carers about smoking did not appear to be an issue unique to Aboriginal carers and patients, but a factor for any child-carer dyad.

*It [screening for smoking] happens infrequently. I’d only take specific smoking history in the setting of recurrent respiratory infections and illnesses like asthma, bronchiolitis, and CSLD [*chronic suppurative lung disease*]. The other risk factors I might take history is if there are other problems such as growth faltering and breastfeeding baby, sometimes smoking might affect supply…or when I have a very small baby, I will ask about smoking during pregnancy. Certainly, when there are no other specific risk factors, I wouldn’t routinely screen for smoking. HSMR 3*

*See if they have come in maybe with something else maybe their foot is sore but then I can tell that the parent smokes, I don’t know how I would go there and be like you need like can we talk about smoking cessation? They will be like - why? (laughter). Because you know the child is not here for that, whereas the kids that come with respiratory issues, I am like all right that is a leeway, but yeah I have not even thought of doing that. HS 4*

Some health staff, however, felt that the culture of the ward and lack of systems to support routine screening for smoking status were partly behind the low prioritisation of smoking cessation support. Health staff mostly attributed low prioritisation of smoking cessation to busy workloads; however, there were indications that smoking cessation was not prioritised even during times of reduced activity. In contrast, other staff argued that time could be made regardless, as this was also part of family-centred care.

*When I come on shift, I notice that there is a lot of sitting around and not a lot of working with the families. We should be sitting with families, educating - whether it’s with smoking or anything – we should have time in the day to sit there, even if it’s only for ten minutes per patient, just to sit there and just have a chat because quite often you’ll find out things that they need help with. HS 10*

*I do think it is important that we do address these issues and make it a priority, but sometimes it is not the most important thing at the time. HS 1*

### Lack of systems and procedures to provide support for screening for smoking

Staff reported that there was a lack of systems and procedures to support screening for smoking. Health staff knew the importance of smoking but may not want to do anything that went against common practice, even if their actions would be in line with evidence-based medical care.

*I don’t know if I have any support cos I’m just going in and talking to a parent, but if I know that it is a thing that is established in the unit and that everyone has the same goal. If everyone is doing the same thing or required to do the same. I wouldn’t feel weird about it cos I will be saying oh yeah we planned and that’s what we are doing. HS 4*

Health staff wanted a standard procedure or protocol to follow so that they could justify why they were raising the topic of smoking. If it was part of their standard procedure, they would feel more confident in their approach, rather than it being perceived as singling people out and discriminatory. They suggested that including screening for smoking in the admission paperwork would normalise talking about and increased offers of smoking cessation support.

*We should have proper guidelines, and protocol…this is the way, like the best approach, so we know what to do. Everyone will be on the same page. HS 7*

*If we establish it from the beginning, rather than like budging in the middle of the admission and start talking about smoking, then it’s a thing that wasn’t mentioned at the beginning - then someone will start thinking this was never a problem from the beginning, why is it a problem now? HS 4*

*If it’s included on the admission pack maybe it could prompt the nurses to ask that question. Then from there they can identify maybe how they can introduce the cessation part of it to the parent or suggest ways which can minimise that SHS exposure. HS 2*

Standardisation of smoking cessation screening of carers on admission was perceived by staff as having the potential to improve compliance and overcome barriers like time constraints.

*If you have a standardised thing, maybe we will find the time and like spend time and do it. But if it’s just like part of the care, you just - ok, when you get a chance to do it, you do it. HS 7*

Reflecting the lack of standardisation, smoking cessation support was not adequately recorded in the clinical records. Staff did not know what conversations might have already occurred with the carer. Record keeping would help to guide the team as to where a carer was at in terms of their readiness to quit.

*I don’t have a sense where they are at; they might feel like they have already been interrogated in the past by other people, so I don’t know where there are at from that point of view as well, whether they will be receptive. HSMR 3*

Staff also reported that there was a lack of clear procedures about what to do next after screening for smoking. When collecting information about carers’ smoking status, health staff did not perceive that their actions triggered a referral pathway to ensure that the carer received appropriate assistance. Health staff therefore sometimes omitted smoking screening and discussing cessation because they did not see the benefit of this to the carers.

*If I did screen for it, I’m not sure what the next step will be whether it will be just information that we collect but would it necessarily enact a plan. It does not trigger any particular response. HSMR 3*

*I’m not sure if it gets picked up by nursing staff per se, or it probably does, but they don’t necessarily act upon it because quite often you’ll see the parents going outside for the cigarette, or just letting staff know and going out to have a cigarette when they’ve got a child with a respiratory illness, then staff will quite often just say, “Yep. No worries. See you when you get back”. HS 10*

### Unclear systems for providing more detailed cessation support to carers

Some health professionals did not think they should be providing smoking cessation support as part of their role. Some nurses perceived their role as only raising issues about smoking and identifying carers who were smokers for a referral to the respiratory educator, They did not view having in-depth discussions with carers as their responsibility.

*We don’t really get to talk about it as nurses, but we know that if someone comes with a chest infection maybe then part of the care will involve a parent or child that will have been reviewed by the respiratory educator HS 3*

*There is actually maybe one or two ladies whom I have spoken to about quitting of smoking, and they said “sister yes I really want to quit”, then I said ok let me call respiratory educator for you. Not me sitting down and talking to them because I’d have spent maybe a good 10 to 20 minutes explaining to them that process than when he comes with all the options that he can do; the patches and all those things. HS 3*

There was also a perception among staff that the respiratory educator was the only person with clinical expertise to provide smoking cessation interventions. Previously, the respiratory educator had provided smoking cessation education and advice, as well as proactive follow up with patients’ primary care and specialist clinics for patients with illnesses such as pneumonia and bronchiectasis. However, at the time of the study, the respiratory team resources were being reduced, so the respiratory educator was only able to offer general smoking cessation advice and no longer had the capacity to offer a complete smoking cessation service with individual patient follow up. This was not being communicated to other staff, with the result that health staff in the paediatric ward did not seem to be aware of the changes, and still followed past procedures when the respiratory educator would attend to all referrals. These changes have created role conflict whereby no one felt it was their responsibility to provide smoking cessation support.

Some health staff, however, felt that because of the intimate knowledge they have of the patient and family, it should be their responsibility to talk about smoking. There was also a perception that this advantageous position and rapport was underutilised, and there was capacity for them to contribute more to smoking cessation. Similarly, some doctors felt that nurses were better placed than medical staff to provide smoking cessation.

*The respiratory educator, they don’t know the family. They don’t know the parents. They don’t know their situation. As a nurse, we know them better [than any other health staff]. So, I think we can do more than talk to just another respiratory educator. They can get involved, and they can introduce – like they can refer to like a respiratory educator if they have a protocol, like guidelines or any program. They can introduce that. But mainly, I think it’s the nurses’ thing. HS 7*

Other staff suggested that it was only feasible for health staff to deliver brief interventions, where they simply asked about smoking, and then set up the connection to external and more comprehensive services, such as primary care.

*I think it’s a process that needs a lot of follow-up and I don’t think paeds is in a position to do it for the parents of their patients. I think we can talk generally about using patches and so on, but actually prescribing them and following it up, I don’t think should be the role of paediatrics. Because we have a lot of other things that would be very time consuming, I don’t think starting the conversation is an issue at all. HSMR 1*

*It could be a role for primary health care I think that’s probably an organisation that should be taking it up. There is a lot of educational things out there already. HSMR 1*

Other staff however felt that having trained additional staff, preferably Aboriginal, with knowledge of smoking cessation employed within the hospital system would be more effective in alleviating time constraints for both acute care and primary care services.

*If there was even something like a health promotion officer who had multiple skills like drugs and alcohol and cigarettes all those sorts of things as part of a role. Then I think that would be opportunistic things to do in a hospital, but often this stuff is delegated or transferred to primary healthcare providers [e.g. general practitioner or maternal & child health nurses] in the community but they too have specific things and time constraints that they have to meet. So, it probably is a bit of a gap. HSMR 3*

*I would have Aboriginal health workers that have a special interest in smoking cessation to come and work with families, with ready access to all of the resources that they needed. So, you know, they would be able to determine and provide the appropriate strength patches; or gum; or whatever was necessary. And make a follow-up appointment with that person to see how they’re going; or facilitate them signing up to the QUIT [line] if they were wanting to do that. HSMR 2*

### Skills gaps and training needs

Health staff felt uncomfortable about not having knowledge and skills to address smoking, and most were unaware of the language to use when asking about smoking. Other staff stated that they lacked knowledge about nicotine replacement therapy (NRT). As a result, they lacked the confidence to have in-depth conversations with carers.

*I’ve not been taught how to do that. I don’t have the skills of what language to use for short interventions against smoking. HSMR 5*

*A lot of the paediatrics doctors aren’t confident to write it up, either, you know. Because they’re used to paeds stuff, you know, and not used to writing up nicotine. HSMR 7*

The majority of staff stated that they had never received any training about smoking cessation brief interventions and NRT and felt that they would benefit from further training. Health staff suggested that a suite of educational programs and training will improve confidence for providing education, as they would have better understanding of the therapeutic actions and adverse effects of NRT.

*I think more education, not necessarily on the actual topic, but on the delivery part of it. Because even if sometimes you know things, but you don’t know how to say them right, so you try not to make the person not feel judged. HS4*

*I think if all health workers, especially nurses, who are spending a lot of time with the carers and who have opportunities to talk to the carers should be given more education around how to approach, you know, as you said before. How to approach or how to do this questioning, how to approach a mother who is a smoker, what sort of questions you need to ask and how to put this information forward to this—in front of the mother. HS2*

Health staff were unable to offer NRT to support carers who wanted to quit smoking, as carers are not admitted patients and there was no funding or other support systems in place. Rather than offer fragmented care, health staff preferred to refer carers to the respiratory educator who was able to offer holistic interventions.

*When he comes, he comes with all the options that he can do the patches and all those things, actually maybe explain better than me like when you stop smoking you don’t just stop and don’t get anything. There is this which will help you to quit …you know which I don’t really have information maybe if I had that information or if I had those resources I’d explain as well as I go. HS 2*

*If I have the resources the respiratory educator has, if I do not have to refer this person to a respiratory educator to get the resources. If I can give her a nicotine patch myself, or, you know, explain to her more and if she’s willing to do this, you know, I could do it with confidence and more enthusiasm. HS 3*

## Discussion

We found that smoking cessation was a low priority on the paediatric ward, similar to other research within paediatric settings where the presenting illness and other risk factors took precedence over the latent effects of smoking. Time pressures and lack of training were also barriers to screening for smoking and providing cessation support, in both our study and previous research [10, 15]. However, there were opportunities to increase smoking cessation support through improvements to systems and policies for screening and referral of carers who are smokers.

Paediatric units and other settings which have successfully implemented carer smoking cessation support have used a systematic approach, which incorporates guidelines and clinical record-keeping systems (CRS) with embedded screening tools and clear referral pathways [21–23]. These systems include mandatory questions about smoking which must be completed before clinicians can progress to the next section. These compulsory systematic tools for all routine admission assessments increased smoking cessation screening rates compared to units that, like the ward in our study, had staff who limited screening for smoking to carers of patients they perceived as high risk [21–23].

The participants in our study, in line with other research, also said that screening tools provided prompts and cues to remind staff to screen for smoking [24, 25]. Screening tools could also provide specific guidance to help health staff frame discussions about smoking cessation with Aboriginal carers [15]. This guidance may assist with dealing with challenges we previously identified in cross-cultural communication between Aboriginal carers and non-Indigenous health staff, as well as issues identified in this paper [15]. CRS with embedded screening tools have been shown to provide a framework for standardising and normalising communication about smoking cessation. CRS can also provide clear referral pathways and action plans to guide staff on smoking cessation support [24, 25]. Some studies have expressed caution that screening tools could become prescriptive tick boxes that may not produce any measurable outcomes [24]. Careful training of staff could mitigate this risk when implementing a new screening tool.

Participants in our study were keen to screen carers for smoking, but only perceived their role as identifying and referring smokers to specialist quit support. A study of primary health staff in the United Kingdom demonstrated that staff compliance with smoking screening procedures increased with the introduction of clear referral pathways and action plans [26]. The systematic approach ensured that prompt referrals were routinely made for assistance with smoking cessation as would have been done for other risk factors [26]. Participants in our study were demotivated to undertake these processes due to lack of evidence of meaningful outcomes for carers. Introducing systems that show outcomes may assist with increasing motivation.

In our study, smoking cessation advice was often restricted to children with respiratory conditions, as in similar research in a US paediatric hospital [22]. Staff in our study often referred to time and resource constraints as reasons for not screening and providing cessation advice and support to all carers and needed to visualise a direct link between a child’s health and smoking for cessation support to become a sufficient priority to be offered. Similar issues have been reported in various health care settings [21–23]. In both our study and previous research, many health staff had a perception that smoking cessation support was time consuming and should be a separate role from daily routines, which contributes to it being systematically omitted from routine practice [24, 25]. Midwives, who are in a similar position to paediatrics staff as they provide cessation advice to women in part to protect their child, have also been found to perceive that addressing smoking cessation inadvertently replaced other more important tasks [24, 25, 27].

Low prioritisation of carer smoking cessation support among our health staff also seemed to be associated with lack of knowledge and skills. As in other settings. participants in our study stated they did not have sufficient existing knowledge [10], or adequate opportunities to access smoking cessation training [24, 25, 27]. This was important, as health staff who received training have been found to be more likely to carry out smoking assessment and provide cessation support, compared to their untrained counterparts [28, 29]. A small Australian study with general practitioners who provided care to adolescent patients further demonstrated that trained health staff not only provided smoking cessation to their patients but also used a holistic approach to engage patients’ carers to quit smoking [30].

There is growing consensus that smoking cessation should be a fundamental component of basic training for all health professionals, and that educational institutions should be looking at reviewing the programs offered to students during their formative training years [31]. To address this training gap among practitioners already in the workforce, professional organisations and government authorities could work in tandem with health institutions to increase awareness and accessibility to smoking cessation training for health professionals [24, 31].

There was a perception among our staff that smoking cessation programs were more aligned to adult medicine than paediatrics. As a result, paediatric staff – similar to findings with midwives were not often offered training in smoking cessation and did not actively seek training opportunities [24, 27]. Undergraduate degrees do not cover, or give a low priority to, smoking cessation [31]. The gaps in knowledge were also exacerbated by staff lack of awareness about existing opportunities for professional development once in the workforce, such as online smoking cessation training [24, 25, 32]. Providing training will likely enhance staff knowledge and create awareness, and also increase referrals to both Quitline and cessation services provided by general practitioners and other primary health services. These added referral options could address some of the concerns about funding, time constraints and the impact of reduced availability of respiratory services to the paediatric ward. Referral pathways and action plans could be incorporated in the CRS to simplify smoking cessation and improve engagement of health staff.

Brief interventions and NRT are key components of all smoking cessation programs, including hospital-based programs for adult inpatients and for carers of paediatric patients [10, 12–14, 33, 34]. Our participants, similar to health staff in other research [10] were not confident in prescribing NRT, which they considered out of scope of practice for most paediatric doctors. There were also systemic barriers to providing NRT for carers such as funding, monitoring and follow-up of carers, as they were not admitted patients. Modified and shorter versions of brief interventions replacing the 5As with the 3As (Ask, Advise, Arrange) or Ask, Advise, Help (AAH) models are currently being used in Australia and the UK [26, 35]. Training staff in using these shortened versions may assist more use of brief interventions in daily practice and would work well with the systematic changes proposed.

### Strengths, limitations, reflexivity, rigour and validity

The first researcher came to the study as somebody who was immersed in the context, with pre-existing ideas and worldview, which might have influenced the analysis and interpretation [36]. The positioning of the first researcher was a strength as she had good knowledge of the systems and useful resources in which to find information [20, 36], as well as providing easy access to the participants. Participants might have found it easier to share their experiences with a researcher who they believed understood their lived experiences and empathised with the challenges they faced in their day-to-day practice. The researcher used the back-and-forth process of interviews between carers and health staff to clarify themes and to check back for common understanding. This direct communication with Aboriginal carers about their experiences increases the validity of our study.

Conversely, a potential weakness is the role of the first researcher may have influenced the nature of researcher and participant relationship, which then determined what information participants were willing to share [36–38]. This was mitigated by checking back with participants and colleagues about the emerging ideas, as well as by the involvement of the other authors familiar with the research who could provide different perspectives. The study was also limited by not having an Aboriginal researcher and co-author to guide the data analysis. However, the researcher presented preliminary findings of this study to a forum with Aboriginal academics and Elders, which provided an opportunity for critical feedback to strengthen the validity and rigour of our findings by seeking clarification from a cultural perspective. The Standards for Reporting Qualitative Research (SRQR), and Consolidated criteria for reporting qualitative research checklist (COREQ) were used to strengthen the rigour of this article [38, 39].

## Conclusion

This paper addresses an important child health issue and may be relevant to many health staff working in paediatric wards, especially in hospitals with large numbers of Aboriginal and other Indigenous patients in Australia, North America and New Zealand. Screening for smoking and smoking cessation support within our paediatric ward was a low priority, despite the potential benefits of such initiatives for children currently exposed to SHS. The key barriers could be overcome with improved systems to support screening for carer smoking and cessation support, and improved training of paediatric staff in smoking cessation.

## Data Availability

The datasets generated and analysed during the current study are not publicly available due to institutional data sharing policy but can be made available from the corresponding author on reasonable request.
